# Learning, understanding and the use of information technology: a survey study among primary care physician trainees

**DOI:** 10.1186/s12913-019-4615-y

**Published:** 2019-10-22

**Authors:** Michel Wensing, Barbara Paech, Catharina Roth, Simon Schwill

**Affiliations:** 1 Department of General Practice and Health Services Research, Heidelberg University Hospital, Marsilius Arcades, West Tower, Im Neuenheimer Feld 130, 69120 Heidelberg, Germany; 2 Heidelberg University, Institute for Computer Science, Im Neuenheimer Feld 205, 69120 Heidelberg, Germany

**Keywords:** Physician behaviour, Primary care, Information technology

## Abstract

**Background:**

User understanding of information technology systems (IT-Systems) is a prerequisite for their use. This study aimed to explore how primary care physician trainees learn, understand and use IT-Systems.

**Methods:**

A paper-based survey study among 301 primary care physician trainees in Baden-Wuerttemberg, Germany, was performed. The questionnaire included measures of understanding and use of nine specific system features, five possible learning strategies, a validated scale for affinity for technology interaction, and five individual and three practice characteristics.

**Results:**

The sample comprised 94 respondents (31.6% response rate). Between 3.2 and 59.6% said to know specific systems features well; between 13.8 and 42.6% expressed a wish to know more about specific system features. The predominant strategy for learning system features was explanation by others: 51.7 to 66.7% had applied this strategy to learn the features. Between 18.6 and 41.4% had learned the features by trial and error. A better understanding of system features was associated with the use of a trial and error strategy for learning system features (beta = 0.260, *p* = 0.012). The use of a greater variety of learning strategies was associated with higher affinity for technology interaction (beta = 0.215, *p* = 0.037).

**Conclusion:**

The study suggests that many physicians need a better understanding of IT-Systems. The role of manuals, online resources and courses in learning IT-Systems seems limited. The new generation of primary care physicians seem to learn features of IT-Systems through explanation by others and trying in their ambulatory practices. The relevance of IT-Systems in healthcare is high, but physicians need more support in learning to use system features.

## Background and significance

A survey among 9196 primary care physicians from 31 countries in the years 2012 and 2013 found that 99.7% of them used computers in their practice and 87.8% had internet access [1]. Nevertheless, physicians varied substantially in whether and how they actually used the various features of the information technology systems, such as those for documentation or exchange of information on individual patients. Many factors are associated with the actual use of information technology systems by physicians [1, 2], but an obviously relevant factor is the physicians’ understanding of specific system features. Having knowledge of information technology systems was found to be associated with ease of use and perceived usefulness, and ultimately with work-related performance in previous research [3]. Therefore, a better insight into the physicians’ understanding of information technology systems is relevant for the quality and outcome of healthcare delivery as well as for the work experience of health professionals.

A range of strategies is available for learning to use information technology systems, including (in order of decreasing use among computer users generally): trial and error, informal help by family and friends, manuals and online tutorials, informal help by co-workers, and the attendance of courses [4]. A more recent study on different kind of products confirmed that only 25% of the users read the manual [5]. Education and training can effectively enhance the use of information technologies systems by physicians [6]. Nevertheless, there is limited insight into the determinants and mechanisms of effective learning of information technology systems by health professionals.

A possible determinant of learning, understanding and use of information technology systems is the physicians’ affinity for interaction with technology, which can be measured by a questionnaire and was found to vary substantially between individuals outside of healthcare [7]. From a psychological self-regulation theory perspective, the affinity for technology interaction is the individuals’ tendency to approach or avoid technical systems. It can be expected that a high affinity for interaction with technologies is associated with a tendency to explore information technology systems actively, using a trial and error learning strategy. Alternatively, it may encourage the learning of information technology systems generally, and not necessarily the use of any specific learning strategy.

The presented study focused on physicians in the vocational training specializing in primary medical care (general practice/family medicine), because they are the new generation of practising primary care physicians in the coming decades and because they may differ from older generations in their use of information technology systems. Physician trainees see patients in regular one-on-one sessions, but their performance is monitored and supervised by experienced physicians.

### Objectives

Our aim was to explore how primary care physician trainees learn, understand and use information technology systems in primary care practices. Beside a description of these behaviours, we explored the associations with the physicians’ affinity for technology interaction.

## Methods

### Study design

A cross-sectional survey was conducted in November 2018, after having received the ethics approval of the medical ethics committee of the Medical Faculty of Heidelberg University (S600/2018). The survey was announced and supported by the coordinator of the vocational training program. There were no financial incentives for participation in the paper-based survey. A generic reminder was sent out 2 weeks after the initial mailings by email by the coordinator of the vocational training program.

### Study population

Eligible were physicians who were registered in the vocational training program for primary care in Baden-Wuerttemberg (KWBW Verbundweiterbildung^*plus*^) [8], except those who could not yet be expected to have experience in ambulatory practices. The vocational training program, which was only a few years old at the time of this study, is open to recently graduated physicians (who tend to be younger than 30 years of age) as well as to experienced (and older) physicians, who practice in other disciplines than family medicine (e.g. as specialist in internal medicine).

### Measures

The questionnaire (Additional file 1) had a total of 5 sections. Section A focused on demographic and practice characteristics: gender, age (in categories), number of different IT-systems used, participation in vocational training (fulltime/ less than fulltime), year in vocational training (1 to 5), type of patient-records (completely, largely, not computerized), location of practice (city centre, urbanized area, rural area), type of practice (single-handed, group-practice, health centre, other). Descriptive data are presented in Table 1. Section B of the questionnaire comprised the Affinity for Technology Interaction (ATI) Scale, a validated questionnaire of 9 items with a balanced 6-point answering scale ranging from completely agree to completely disagree [7]. Descriptive data are presented in Additional file 2.

**Table 1:** Description of study population (*n* = 94 physicians)

		N (%)
Individual characteristics		
1	Gender	
	Women	68 (72.3%)
2	Age in years	
	25–29	12 (12.2%)
	30–34	38 (40.4%)
	35–39	18 (19.1%)
	40–44	6 (6.4%)
	45–49	10 (10.6%)
	50+	10 (10.6%)
3	Number of IT-systems used	
	1	81 (86.2%)
	2	9 (9.6%)
	3	3 (3.2%)
	4 or more	1 (1.1%)
4	Participation in vocational training	
	50–99% of fulltime	44 (46.8%)
	100% (fulltime)	50 (53.2%)
5	Year in vocational training	
	1	1 (1.1%)
	2	4 (4.3%)
	3	13 (13.8%)
	4	35 (37.2%)
	5	39 (41.4%)
	Recently completed / unknown	2 (2.2%)
Practice characteristics		
1	Type of patient records in practice	
	Completely computerized	68 (72.3%)
	Mainly computerized	23 (24.5%)
	Mainly paper-based / other	3 (3.2%)
2	Location of primary care practice	
	City centre	38 (40.4%)
	Urbanized area	42 (44.7%)
	Rural area	14 (14.9%)
3	Type of practice	
	Single handed	26 (27.7%)
	Group practice	58 (61.7%)
	Health centre	8 (8.5%)
	Other	2 (2.1%)

The remaining sections (C, D and E) of the questionnaire focused on use, learning, and understanding of IT-systems respectively, focusing on nine specific system features: the management of medical patient data, an overview of the medical data of a patient, writing of letters to other physicians, ordering of treatments, the interpretation of medical data (e.g. test results), administrative coding for reimbursement, provision of patient information in consultations, an overview of practice data (e.g. prescriptions), quarterly overviews and cost statements. Table 2 summarizes the descriptive data for sections C and E; Table 3 provides description data for section D.

**Table 2:** Use and understanding of system features (*n* = 94 physicians)

	Daily use^a^	Know what to do to use these features^b^	I can use these features but would like to be better^b^
1 Management of medical patient data	89 (94.7%)	48 (50.1%)	33 (35.1%)
2 Overview of medical data of a patient	88 (93.6%)	52 (55.3%)	35 (37.2%)
3 Writing of letters to other physicians	87 (92.6%)	56 (59.6%)	26 (27.7%)
4 Ordering of treatments	84 (89.4%)	46 (48.9%)	35 (37.2%)
5 Interpretation of medical data (e.g. test results)	78 (83.0%)	48 (51.1%)	32 (34.0%)
6 Administrative coding for reimbursement	75 (79.8%)	13 (13.8%)	40 (42.6%)
7 Provision of patient information in consultations	69 (73.4%)	39 (41.5%)	27 (28.7%)
8 Overview of practice data (e.g. prescriptions)	26 (27.7%)	8 (8.5%)	22 (23.4%)
9 Quarterly overviews and cost statements	22 (23.4%)	3 (3.2%)	13 (13.8%)

^a^Answering categories were: daily use; incidental use; no use. ^b^Answering categories were: I know what to do to use these features; I can use these features but would like to be better; I know something and can use these features slowly; I know little; I know nothing

**Table 3:** Learning of system features (*n* = 94 physicians)

	Learning strategies used (N, %)*2					
*1	Manual	Online source	Course	Others explained	Trial and error	Other ways
1 Management of medical patient data (*n* = 94)	5 (3.6%)	0	2 (1.4%)	84 (60.0%)	47 (33.6%)	2 (1.4%)
2 Overview of medical data of a patient (*n* = 94)	3 (2.3%)	0	1 (0.8%)	76 (59.4%)	44 (34.4%)	4 (3.1%)
3 Writing of letters to other physicians (*n* = 94)	2 (1.6%)	0	2 (1.6%)	86 (66.7%)	37 (28.7%)	2 (1.6%)
4 Ordering of treatments (*n* = 93)	3 (2.3%)	0	1 (0.8%)	83 (64.8%)	40 (31.3%)	1 (0.8%)
5 Interpretation of medical data (e.g. test results) (*n* = 92)	3 (2.5%)	0	1 (0.8%)	65 (54.2%)	46 (38.3%)	5 (4.2%)
6 Overview of practice data (e.g. prescriptions) (*n* = 77)	2 (2.2%)	0	0	56 (60.2%)	21 (22.6%)	14 (15.1%)
7 Provision of patient information in consultations (*n* = 90)	3 (2.6%)	0	1 (0.9%)	60 (51.7%)	48 (41.4%)	4 (3.4%)
8 Administrative coding for reimbursement (*n* = 92)	3 (2.4%)	2 (1.6%)	3 (2.4%)	81 (65.9%)	32 (26.0%)	2 (1.6%)
9 Quarterly overviews and cost statements (*n* = 71)	2 (2.3%)	0	0	51 (59.3%)	16 (18.6%)	17 (19.8%)

*1 Figures lower than *n* = 94 indicate that not all physicians answered the question, *2 multiple answers possible, percentages are reported using denominator as sum of all the answers including duplicates

The most frequently used information technology system in the practice (German: ‘Praxisverwaltungssystem’) was asked for and the questions on the use in section C were focused on this system. The questions on the use of these features (section C in the questionnaire) had three answering options: daily use; incidental use; no use. In section D of the questionnaire, five strategies for learning (read manual, use online sources, attend a course, explanation by others, trial and error, other strategies) were specified and related to each of the nine system features, requesting to indicate whether these were used (yes/no answering format). In section E, the degree of understanding of each the nine system features was documented, providing five answering options: I know to use; I can use it but would like to be better; I know something and can use it slowly; I know little; I know nothing.

At the end the questionnaire contained two open-ended questions on improvement suggestions for faster learning and better use. The questionnaire was informed by literature review and an educational project of informatics students at Heidelberg University in 2018, in which the students interviewed physicians on their use of IT-systems. We piloted a draft-version of the questionnaire with a few colleagues, which led to some modifications and additional explanations.

### Data-analysis

Descriptive statistics were obtained. Then aggregate measures were constructed for further analysis: a) a mean value on the 9 items of the ATI scale, b) a mean value on the degree of understanding on the 9 system features, c) a count of the number of system features, which were used daily, d) a count of the number of system features, for which a specific learning strategy was used, e) the number of different learning strategies used across system features. The answers to the open questions were categorized in a content analysis.

**Fig. 1:**
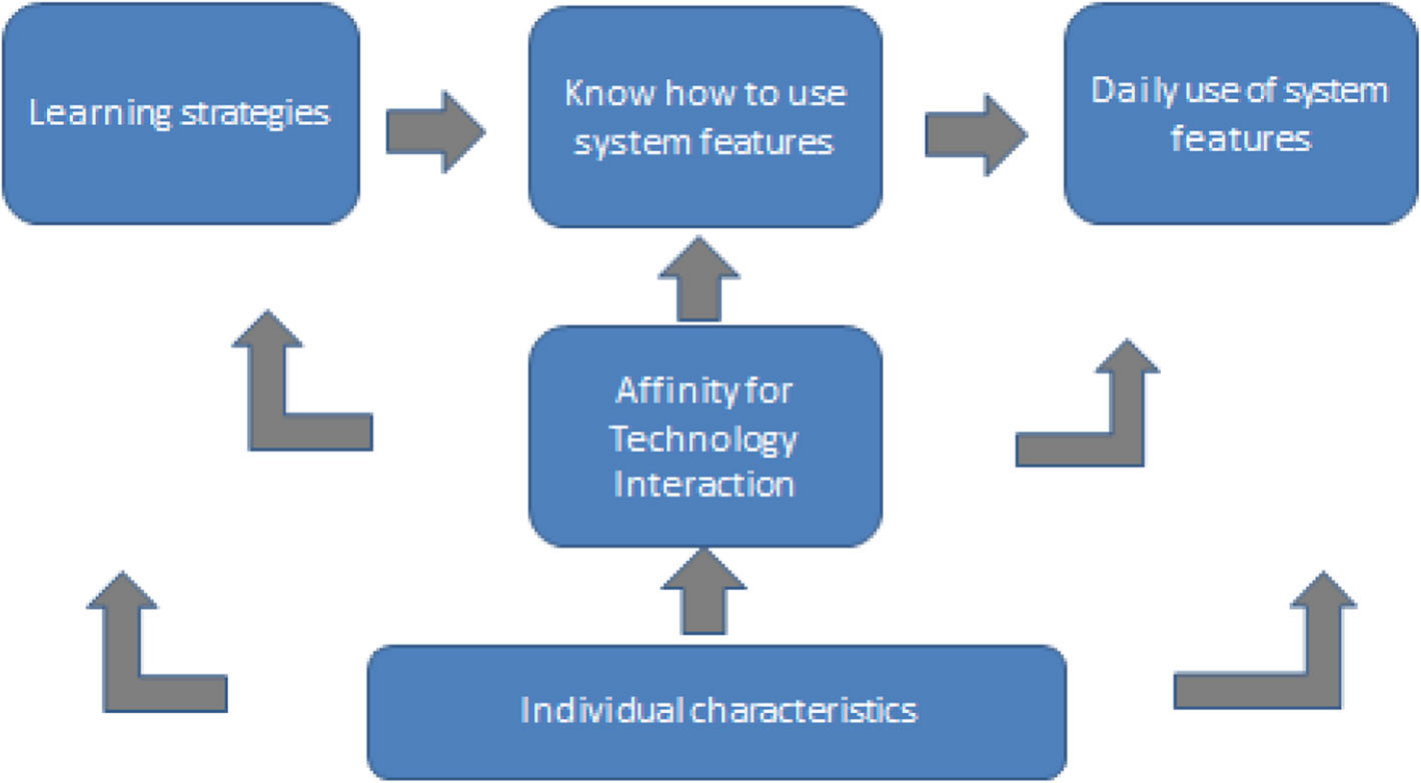
Tentative conceptual model to guide data-analysis

Several bivariate linear regression analyses were applied to explore the impact of predictors on the five aggregate measures. In the absence of previous research, we used our knowledge of the target group and setting to speculate about possible associations. To guide data-analysis, we developed a tentative conceptual model (Fig. 1). The conceptual framework (Fig. 1) was a heuristic tool rather than a set of strong hypotheses.

The tentative conceptual model suggests that learning strategies influence the understanding of information technology systems, which then influenced the actual use of these systems. Research on continuing education of physicians showed that passive participation in educational meetings (e.g. courses and conferences) has limited effect on the physicians’ behaviours and healthcare processes [9]. Active involvement in the education is supposed to be crucial to the effectiveness of educational programmes [10]; ‘trial and error’ (or experimenting) seems a good indicator of active involvement in the learning of an IT-system. On the other hand, the use of trial and error as a single learning strategy is unlikely to result in comprehensive understanding and use of the IT-systems [11]. Summarizing, it may be assumed that a combination of participation in formal education (e.g. using a manual or taking a course) and active participation (e.g. trying the system) results in the highest understanding and use of IT-system features.

Affinity for technology interaction (ATI scale) may influence learning strategies, the understanding and use of systems, although we did not have specific hypotheses on these associations. For instance, a strong affinity for technology interaction may be specifically related to the use of a trial and error learning strategy, or simply encourage the learning of IT systems generally. The aggregate measure for the use of system features was regressed upon the aggregate measure of the understanding of system features and the ATI scale. The aggregate measure for the understanding of system features was regressed upon the aggregate measures of the learning strategies and the ATI scale. The aggregate measures of learning strategies were regressed upon the ATI scale. All dependent factors, and the ATI scale, were also regressed upon the 9 individual and practice characteristics. Predictors with bivariate significant effects were included in combined regression models, but these were considered as highly tentative given the sample size.

Sensitivity analyses concerned a) alternative aggregate measures for use and understanding of system features, which did not include two features that are typically not used daily (overview of practice data and quarterly overviews), b) an aggregate measure, which linked a learning strategy and the understanding on the level of specific system features. None of these showed substantially different results from the main analyses (results not presented). All analyses were performed in SPSS 25. *P*-values < 0.05 were considered significant.

Qualitative data was analysed using quantitative content analysis. In a first step, answers to the two open-ended questions were gathered and merged into one document. In a next step, answers were coded based on how many times relevant information occurred in the data (frequency-based purpose). Initial codes were discussed by the principal investigator and the third author. The quantified codes were then statistically analysed to address the research questions. Software for qualitative analysis was not used.

## Results

### Description of the sample

Of a total of 301 eligible physicians, 95 responded (31.6% response rate). One physician had never used an information technology system in an ambulatory practice before and was excluded, leaving 94 respondents. Table 1 presents the descriptive characteristics of the sample. Of the total 72.3% (*n* = 68) were female. About half of the respondents (52.6%) were 34 years or younger. A vast majority of the respondents (86.2%) indicated that they had used only one information technology system. Only just over half of the respondents (53.2%) reported that they participate fulltime in vocational training, the other part (46.8%) indicated that they attend vocational training between 50 and 99% of fulltime. A large proportion of participants are in year 5 (41.4%) or year 4 (37.2%) of vocational training. Less than 20% are in year 3 (13.8%). The smallest part is in year 2 or 1 of vocational training (4.3 and 1.1%, respectively).

Almost three quarters of the respondents (72.3%) stated that their practice documented completely computerized, a quarter (24.5%) stated that their practice documented mainly computerized, and a few (3.2%) said that they mainly documented paper-based. Almost half of the primary care practices are located in urbanized areas (44.7%), the other primary care practices are located in the city centre or in rural areas (40.4 and 14.9%, respectively). More than half of the primary care practices are led as group practice (61.7%). Almost one third were managed by a single physician (27.7%), only a small part of the primary care practices was embedded in a health centre (8.5%).

The affinity for technology interaction varied substantially, with 22.4 to 55.6% indicating high affinity on items of the ATI Scale and the others indicating low affinity (Additional file 2).

### Use and understanding of system features

A large majority of the participating physicians (73.4 to 94.7%) stated that they used 7 out of 9 features on a daily basis (features 1–7 in Table 2). About half of the respondents (between 41.5 and 59.6%) indicated that they knew what they have to do to use these features properly. Nevertheless, even though a large majority of the participating physicians had to use most features (1 to 7) on a daily basis due to their working routine, around one third of the participants (between 27.7 and 42.6%) stated that they would like to understand these seven features better with the highest percentage for feature 7. The exception is the feature for administrative coding (feature 7), which was called “known” by 13.8% only. The remaining two system features (making overviews of practice data and quarterly overviews, features 8 and 9 in Table 3) were used less frequently on a daily basis (27.7 and 23.4%, respectively). They were well understood by less than 10% of the physicians. Some physicians (23.4 and 13.8%, respectively) wished to know more about these two features.

### Learning strategies (multiple answers were possible)

The predominant strategy for learning system features were explanation by others: 51.7 to 66.7% of the physicians had used this strategy to learn specific system features (Table 3). Second, relatively many physicians (between 18.6 and 41.4%) had learned features by trial and error. The remaining strategies had been used far less often, with less than 5% of physicians reporting their use.

Table 4 provides descriptive data for the aggregate measures, which were used in the regression analyses. On average, 6.6 of 9 systems features were used daily and the physicians’ understanding of these was 3.3 on average on a scale from 1 to 5. The affinity for technology interaction yielded an average score of 3.3 on the scale from 1 to 6. On average, 6.8 features were learned from others and 4.0 were learned by trial and error; on average, 1.9 different strategies were applied to learn system features.

**Table 4:** Descriptive information on aggregate measures

	System features daily used	System features understanding	Affinity for Technology Interaction	Learning from others	Learning by trial & error	Number of different learning strategies used
Number of items	9	9	9	9	9	9x5 = 45
Answering categories	2	5	6	2	2	2
Scale construction method	Count	Average	Average	Count	Count	Count
Theoretical min- max	0–9	1–5	1–6	0–9	0–9	0–5
Observed min-max	0–9	1–5	1–5.83	0–9	0–9	1–4
Mean score	6.6	3.3	3.3	6.8	4.0	1.9
Cronbach’s alpha	–	0.848	0.915	–	–	–

### Associations between factors

The regression analyses (Additional file 2) showed that the *number of system features used* was associated with better understanding of features (beta = 0.253, *p* = 0.014). Female physicians tended to use fewer system features (beta = 0.211, *p* = 0.041). The affinity for technology interaction did not have a direct effect on the number of system features used. In the multiple regression model, the identified effects remained significant.

A better *understanding of system features* was associated with more frequent use of a trial and error strategy for learning system features (beta = 0.260. *p* = 0.012). Understanding of system features was higher among physicians who worked in single-handed practices as opposed to group practices and health centres (beta = 0.206; *p* = 0.047). The expected effect of the ATI scale on overall understanding of system features was not significant, although close (beta = 0.195, *p* = 0.06). The multiple regression model showed the same significant predictors.

*Learning by trial and error* was more frequently used in practices with a lower degree of computerization (practices with partly paper-based medical records) (beta = 0.203, *p* = 0.049). *Learning from others* was more prevalent among physicians who knew (beta = 0.355, *p* = 0.001) and used (beta = 0.232, *p* = 0.024) fewer information technology systems in their practice (e.g. one as opposed to more systems). Only the effect of the number of systems known remained significant in the multiple regression model. None of the learning strategies was predicted by the affinity for technology interaction. However, the *use of a greater variety of learning strategies* was predicted by higher affinity for technology interaction (beta = 0.215, *p* = 0.037).

Finally, the affinity for technology interaction was somewhat higher in male physicians (beta = 0.277, *p* = 0.007) and in physicians in fulltime training (beta = 0.272, *p* = 0.008). In the multiple regression analysis with both predictors, however, these effects did not remain significant.

### Answers to open-ended questions

Forty-seven respondents suggested improvements for a faster learning and 35 for better the use of information technology systems. A majority of them suggested courses or seminars as resource for faster learning (*n* = 24). The physicians also stated that these would help them to use the features better. Twelve participants stated that tutorials or manuals might be useful for faster learning as well as using features better. Introduction by experts, colleagues or educators was mentioned as useful source for faster learning by 10 respondents. Additionally, 7 physicians stated that a structural introduction would also be helpful regarding a better use of features. Only 3 stated that practical support might be helpful in the case of faster learning and none stated that support might have an impact on a better use. Six physicians reported that improving working conditions like for example more time and less effort would be beneficial regarding faster learnability as well as better use of the features. However, a number of the physicians (*n* = 13) stated that improving the information technology itself (e.g. clarity, enhancing user-friendliness, minimising disturbances, activating all features) would help to learn the features faster and therefore help them to use features better.

## Discussion

### Main findings

The large majority of physicians in vocational training for primary care reported to use many features of information technology systems on a daily basis. Their degree of understanding of the features was mixed, with about a third expressing a wish to have a better understanding of system features. Learning from others and learning by trial and error were the predominant strategies for learning the features of information technology systems in ambulatory practices. Manuals, online information, and courses were rarely used. The regression analyses suggested that a high affinity for technology interaction and the use of multiple learning strategies were associated, as well as the use of trial and error and degree of understanding of system features, and a high degree of understanding of the system features and their use. If the tentative conceptual model is correct, the findings may suggest that a high affinity for technology interaction leads to a greater variety of learning strategies, which in this sample often implies trial and error and learning from others. Following the conceptual model, trial and error of system features was found to enhance better understanding of these features, which enhances their daily use in practice. Given the cross-sectional and explorative character of the study, the suggested causality of the associations is tentative.

### Interpretation

Although most of the participants have to use the system features daily, since it is part of their daily working routine, many would like to know how to use them better. Therefore, the number of people who use the features on a daily basis was higher than the number of people who know how to use these features. The limited use of information and education confirms the finding of other research [4]. In other research, however, trial and error was more common than help by others [4]. Interestingly in the open questions on learning improvements, the majority of suggestions related to information and education, while far fewer asked for informal personal help, tutorials or improved interfaces. It should be noted, however, that only a minority of all respondents expressed such suggestions. We suspect that the available courses on information technology are in fact not known or perceived as too expensive.

The findings suggests that information and education can be relevant, but the content and format needs to be considered. Also, physicians may need encouragement and facilitation to take courses and read materials to enhance their computer skills. Despite many studies in medical education, only few studies focussed on the different learning types [12] among physicians in post-graduate training [13, 14]. A typical way of learning in primary care physician trainees has not been identified. A possibility why they do not favour written information or courses for learning about information technology systems is that they do not prioritize this (non-medical) topic and try to save time.

The study found a large variation in affinity for interaction with technology, with overall somewhat lower affinity than in other samples [7]. The mean value in our sample was 3.3 on the answering scale with six categories, while the mean value ranged from 3.6 to 4.4 in the nine samples in the study by Franke et al. [7]. Lower average values were found in samples which seem closest to the general adult population, while the higher values were found in samples of individuals with experience in using computers through training or interest. The sample of physicians seems closer to the general adult population than to computer scientists. The affinity for interaction with technology was not consistently related to individual and practice characteristics. In particular, physician age was not consistently related to the affinity for technology interaction: younger physicians did not have consistently higher affinity. Furthermore, high affinity for interaction with technology was not related to a specific learning strategy, but to the use of larger variety of learning strategies and -nearly significant- to a better understanding of IT system features. Therefore, it seems relevant to explore this concept in future studies among physicians.

This study did not examine whether the use of information technology systems in primary care is associated with better quality, efficiency or outcome of care. Other research has shown that various types of information technology systems can have positive impacts, for instance on the adherence to clinical practice guidelines, the number of medication errors, adverse treatment outcomes and time needed for documentation [15]. While information technology systems probably have an overall positive impact on the quality and outcomes of healthcare, this is not necessarily true for each system feature and for all patients.

### Methodological limitations

The study has limitations, which need to be acknowledged. With the exception of the ATI scale, the questionnaire was newly developed and, although carefully developed and piloted, not separately validated. The cross-sectional study design did not allow for the testing of causality and the modest sample size did not allow for multivariate analyses with robust results. The findings of the study, and particularly the results of the regression analyses, should be seen as tentative. The response rate is average for survey research among physicians, but it may obviously imply selection bias. The generalizability beyond the targeted population of physicians in vocational training in one jurisdiction in Germany is uncertain.

### Implications

There is a need for better understanding of information technology systems among primary care physicians, which would also contribute to the use of more system features. Training and support activities should take into account that many physicians currently learn system features from others and by trial and error. The importance of active learning as well as the role of social interaction has been emphasized by educational science [16]. Teaching by a trainer in the ambulatory practice or in a workshop, directly followed by (supervised) trying of system features, may be a feasible and effective learning strategy for primary care physicians. Nevertheless, there is evidence to believe that there remains a need for structured information and education on information technology systems in ambulatory care [11]. Such training is in fact offered to ambulatory physicians by health insurers and physician organisation in Baden-Wuerttemberg, but these are largely focused on administration for reimbursement. In order to increase the relevance for physician trainees, and thus to improve the quality and outcome of healthcare, it seems crucial to better focus the information and courses on medically relevant topics. The information should also be tailored to the workflows of practising physicians [17].

## Conclusion

The relevance of information technology systems in healthcare is high and increasing, but physicians need more support in learning to use the system features. Although they use information systems on a daily basis, many would like to understand them better. The content and format of manuals, online information and courses on IT systems for physicians needs to be reconsidered as they were hardly used. Trial and error and advice from others were the predominant strategies for learning IT systems, but it is unlikely that these help to achieve the best learning outcome. The affinity for interaction with technology varied widely among primary care physician trainees and may have an impact on learning and understanding of IT systems; future research should examine this more extensively.

## Supplementary information

**Additional file 1.** Questionnaire.

**Additional file 2.** Additional tables.

## Data Availability

The datasets generated and analysed during the current study are not publicly available since the information and consent procedure does not facilitate secondary use of the data.
