# Supplementary material for: Learning, understanding and the use of information technology: a survey study among primary care physician trainees
Source: BMC Health Serv Res. 2019 Oct 22;19:728. doi: 10.1186/s12913-019-4615-y (PMC6805569; doi:10.1186/s12913-019-4615-y)
Supplement: Supplementary file 1 — Additional file 1. Questionnaire. [file 12913_2019_4615_MOESM1_ESM.docx]

**Demographic background information**

*Please tick as appropriate:*

| Please indicate in which age group you are: | - Between 25 to 29 years - Between 30 to 34 years - Between 35 to 39 years - Between 40 to 44 years - Between 45 to 49 years - 50 years or older |
| --- | --- |
| What gender are you? | - Female - Male - Other: _______________________ - Prefer not to say |
| Participation in vocational training | - 100% - 75% - 99% - 50% - 74% |
| How many different Practice Management Software Systems do you know? | - none - Between 1 and 2 - Between 2 and 3 - Between 3 and 4 - More than 4 |
| How many different Practice Management Software Systems have you used? | - none - Between 1 and 2 - Between 2 and 3 - Between 3 and 4 - More than 4 |
| Where is your current primary care practice located? | - City centre - Urbanized area - Rural area |
| Which type of patient records do you use in your current primary care practice? | - Completely computerized - Mainly computerized - Mainly paper-based - Other |
| What type is your current primary care practice? | - Single handed - Group practice - Health centre - Other |
| In which year of vocational training are you in? | - Year 1 - Year 2 - Year 3 - Year 4 - Year 5 |

**B Affinity for technology interaction**

In the following questionnaire, we will ask you about your interaction with technical systems. The term “technical systems” refers to apps and other software applications, as well as entire digital devices (e.g., mobile phone, computer, TV, car navigation).

Please indicate the degree to which you
agree/disagree with the following statements.

|  | | Completely disagree | Largely disagree | Slightly disagree | Slightly agree | Largely agree | Completely agree |
| --- | --- | --- | --- | --- | --- | --- | --- |
| **1** | I like to occupy myself in greater detail with technical systems. |  |  |  |  |  |  |
| **2** | I like testing the functions of new technical systems. |  |  |  |  |  |  |
| **3** | I predominantly deal with technical systems because I have to |  |  |  |  |  |  |
| **4** | When I have a new technical system in front of me, I try it out intensively. |  |  |  |  |  |  |
| **5** | I enjoy spending time becoming acquainted with a new technical system. |  |  |  |  |  |  |
| **6** | It is enough for me that a technical system works; I don’t care how or why. |  |  |  |  |  |  |
| **7** | I try to understand how a technical system exactly works |  |  |  |  |  |  |
| **8** | It is enough for me to know the basic functions of a technical system. |  |  |  |  |  |  |
| **9** | I try to make full use of the capabilities of a technical system. |  |  |  |  |  |  |

**C Using Practice Management Software Systems**

This part of the questionnaire refers to Practice Management Software Systems only used in primary care practices

| **Which Practice Management Software do you use at the moment or did you used the most?** |  |
| --- | --- |
| **How do you use the Practice Management Software the most?** | - Administrative coding for reimbursement - Patient records - Other: __________________ |

**Which of the following Practice Management Software features do you use?**

*Please answer the following questions passed on the experience you gained using this system.*

*Please tick as appropriate:*

| Practice Management Software features | | Daily use | Incidental use | No use |
| --- | --- | --- | --- | --- |
| **1** | Management of medical patient data |  |  |  |
| **2** | Overview of medical data of patient |  |  |  |
| **3** | Interpretation of medical data (e.g. test results) |  |  |  |
| **4** | Provision of patient information in consultations |  |  |  |
| **5** | Ordering of treatments |  |  |  |
| **6** | Writing of letters to other physicians |  |  |  |
| **7** | Overview of practice data (e.g. prescriptions) |  |  |  |
| **8** | Administrative coding for reimbursement |  |  |  |
| **9** | Quarterly overviews and cost statements |  |  |  |

**D Learning how to use Practice Management Software**

How did you learn how to use practice management software’s s (*or how are you learning at the moment*)?

| Practice Management Software features | | Manual | Online sources | Course | Others explained | Trial and error | Other strategies |
| --- | --- | --- | --- | --- | --- | --- | --- |
| **1** | Management of medical patient data |  |  |  |  |  |  |
| **2** | Overview of medical data of a patient |  |  |  |  |  |  |
| **3** | Interpretation of medical data (e.g. test results) |  |  |  |  |  |  |
| **4** | Provision of patient information in consultations |  |  |  |  |  |  |
| **5** | Ordering of treatments |  |  |  |  |  |  |
| **6** | Writing of letters to other physicians |  |  |  |  |  |  |
| **7** | Overview of practice data (e.g. prescriptions) |  |  |  |  |  |  |
| **8** | Administrative coding for reimbursement |  |  |  |  |  |  |
| **9** | Quarterly overviews and cost statements |  |  |  |  |  |  |

*Please tick as appropriate (*[*multiple answers possible*](https://www.linguee.de/englisch-deutsch/uebersetzung/multiple+answers+possible.html)*):*

If you tick „Other strategies“ please explain your learning strategy here:

__________________________________________________________________________________

__________________________________________________________________________________

__________________________________________________________________________________

__________________________________________________________________________________

__________________________________________________________________________________

__________________________________________________________________________________

__________________________________________________________________________________

__________________________________________________________________________________

__________________________________________________________________________________

**E Understanding Practice Management Software**

How well do you understand Practice Management Software Systems regarding knowledge and use of different features?

*Please tick as appropriate*

| Practice Management Software features | | I know nothing | I know little | I know something and can use it slowly | I can use it but would like to be better | I know how to use |
| --- | --- | --- | --- | --- | --- | --- |
| **1** | Management of medical patient data |  |  |  |  |  |
| **2** | Overview of medical data of patient |  |  |  |  |  |
| **3** | Interpretation of medical data (e.g. test results) |  |  |  |  |  |
| **4** | Provision of patient information in consultations |  |  |  |  |  |
| **5** | Ordering of treatment |  |  |  |  |  |
| **6** | Writing letters to other physicians |  |  |  |  |  |
| **7** | Overview of practice data (e.g. prescriptions) |  |  |  |  |  |
| **8** | Administrative coding for reimbursement |  |  |  |  |  |
| **9** | Quarterly overviews and cost statements |  |  |  |  |  |

**What would be helpful to improve learning how to use Practice Management Software Systems and its features quicker?**

__________________________________________________________________________________

__________________________________________________________________________________

__________________________________________________________________________________

__________________________________________________________________________________

__________________________________________________________________________________

__________________________________________________________________________________

__________________________________________________________________________________

__________________________________________________________________________________

**What would be helpful to improve the use of Practice Management Software Systems and its features in practice**

__________________________________________________________________________________

__________________________________________________________________________________

__________________________________________________________________________________

__________________________________________________________________________________

__________________________________________________________________________________

__________________________________________________________________________________

__________________________________________________________________________________

**Thank you for filling in the questionnaire!**
