# Supplementary material for: Learning, understanding and the use of information technology: a survey study among primary care physician trainees
Source: BMC Health Serv Res. 2019 Oct 22;19:728. doi: 10.1186/s12913-019-4615-y (PMC6805569; doi:10.1186/s12913-019-4615-y)
Supplement: Supplementary file 2 — Additional file 2. Additional tables. [file 12913_2019_4615_MOESM2_ESM.docx]

**Additional Tables**

**Table 1. Affinity for Technology Interaction** (n=94 physicians)

|  | High Affinity for Technology Interaction * N (%) |
| --- | --- |
| 1. I like to occupy myself in greater detail with technical systems. | 36 (38.2%) |
| 2. I like testing the functions of new technical systems. | 44 (46.8%) |
| 3. I predominantly deal with technical systems because I have to. | 35 (27.2%) |
| 4. When I have a new technical system in front of me, I try it out intensively. | 48 (51.1%) |
| 5. I enjoy spending time becoming acquainted with a new technical system. | 27 (28,6%) |
| 6. It is enough for me that a technical system works; I don’t care how or why. | 21 (22.4%) |
| 7. I try to understand how a technical system exactly works. | 34 (36.1%) |
| 8. It is enough for me to know the basic functions of a technical system. | 39 (41.5%) |
| 9. I try to make full use of the capabilities of a technical system. | 55 (55.6%) |

**Legend**. * Highest three categories on the answering scale (indicating agreement), except questions 3,6,8 for which the lowest three categories (indicating disagreement) are reported.

**Table 2. Summary of significant effects in regression analysis (reported are bivariate sign. coefficients)**

| **Dependent🡪**  **Predictors:** | IT system use (cat. 1) | IT system understanding | Learning from others (Learn 1) | Learning by trial + error (Learn 2) | Affinity for Technology Interaction | Number of different learning strategies |
| --- | --- | --- | --- | --- | --- | --- |
| Gender | Beta= 0.221 p=0.041 | - | - | - | Beta=  0.277  p=0.007 | - |
| Age | - | - | - | - | - | - |
|  |  |  |  |  |  |  |
| Part-/fulltime status | - | - | - | - | Beta=  -0.272  P=0.008 | - |
| Year in vocational training | - | - | - | - | - | - |
| Number of IT-systems known | - | - | Beta=  -0.355  p<0.001 | - | - | - |
| Number of IT-systems used | _ | - | Beta=  -0.232  P=0.024 | - | - | - |
| Location of practice | Beta=0.200  P=0.053 | - | - | - | - | - |
| Computerization of practice | _ | - | - | Beta=0.203  p=0.049 | - | - |
| Type of practice | - | Beta=  -0.206  p=0.047 | - |  | - | - |
|  |  |  |  |  |  |  |
| IT system understanding | beta= 0.253 p=0.014 | n.a. | n.a. | n.a. | n.a. | n.a. |
| Affinity for Technology Interaction | - | NS beta =0.195, p=0.060 | - | - | n.a. | Beta=  0.215, p=0.037 |
| Learning from others | n.a. --- | - | n.a. | n.a. | n.a. | n.a. |
| Learning by trial & error | n.a. --- | beta=  0.260,  p= 0.012 | n.a. | n.a. | n.a. | n.a. |
| Number of different learning strategies used | n.a. | - | n.a. | n.a. | n.a. | n.a. |
| **Summary of multivariate analysis with sign. predictors** | **Same sign; R2:14.6%** | **Same sign;**  **R2:37,4%** | **Only systems known sign; R2:35,7%** | n.a. | **No sign predictors; R2:32,3%** | n.a. |
